# Occurrence of Trace-Level Antibiotics in the Msunduzi River: An Investigation into South African Environmental Pollution

**DOI:** 10.3390/antibiotics13020174

**Published:** 2024-02-09

**Authors:** Temesgen Zelalem Addis, Joy Tuoyo Adu, Muthukrishnavellaisamy Kumarasamy, Molla Demlie

**Affiliations:** 1 Civil Engineering Programme, School of Engineering, University of KwaZulu-Natal, Durban 4041, South Africa; tommzell@gmail.com (T.Z.A.); kumarasamy@ukzn.ac.za (M.K.); 2 Saveetha School of Engineering, Saveetha Institute of Medical and Technical Sciences, Chennai 600072, India; 3 Department of Geological Science, University of KwaZulu-Natal, Durban 4041, South Africa; demliem@ukzn.ac.za; 4 School of Earth Sciences, Bahir Dar University, Bahir Dar P.O. Box 79, Ethiopia

**Keywords:** antibiotics, emerging contaminant, ecological risk assessment, liquid chromatography-–mass spectrometry, South Africa

## Abstract

The presence of antibiotics in the environmental matrix has raised concerns regarding their risk to the aquatic ecosystem and human health. Surface water, such as rivers, plays a pivotal role in the dispersion and transport of antibiotic residues. The effective monitoring of these contaminants requires investigating their sources and distribution. While numerous studies have been conducted globally to comprehend the emergence, prevalence, and management of these substances, the investigation of therapeutic antibiotics in Africa remains notably underrepresented. Consequently, data regarding these emerging contaminants in the African aquatic environments are scarce, warranting further exploration. This study aims to investigate the occurrence of four specific therapeutic antibiotics—tetracycline, sulfathiazole, penicillin g, and erythromycin—across different seasons in the Msunduzi River, Eastern South Africa. Three sampling campaigns were conducted during spring, autumn, and winter to assess the presence of these antibiotics in the river. Analyte extraction from water samples was achieved through solid-phase extraction, and quantification was performed using liquid chromatography–mass spectrometry. The findings reveal notable concentrations of these antibiotics in the river at locations closest to a wastewater treatment discharge point. Among the antibiotics studied, tetracycline (158.42–1290.43 ng/L) and sulfathiazole (112.68–1151.25 ng/L) were the most frequently detected compounds across the majority of the sampling sites and tributaries of the river. Erythromycin was less frequently detected in the surface water and wastewater effluent but was found to be a risk to algal species within the river. While wastewater effluents represent a significant source of antibiotic contamination in the river, tributaries from industrial areas and informal settlements were identified as continuous sources of antibiotic pollution. Thus, it is imperative to implement appropriate monitoring protocols to mitigate antibiotic pollution in the aquatic environment.

## 1. Introduction

The growing global concern about antibiotic residues in aquatic environments stems from the increasing occurrence of antibiotic resistance in humans [1]. Despite antibiotics’ invaluable role in both human and veterinary medicine, prolonged exposure to trace-level antibiotics may induce biological responses in non-target organisms [2]. Thus, the presence of antibiotics in waterways potentially fosters the development and spread of antibiotic-resistant genes among microbial populations [2].

Antibiotics administered to humans and animals, whether orally or topically, for treating illnesses, disease control, or growth promotion, often find their way into the environment through several pathways. Typically, when administered orally, 70% to 90% of antibiotics are excreted through urine and feces within 8 to 24 h after administration [2]. Consequently, antibiotic wastes from residential areas, including informal settlements, and agricultural and industrial areas end up in aquatic environments, such as rivers, streams, ponds, reservoirs, estuaries, lakes, seas, oceans, and groundwater.

The aquatic environment can become contaminated with antibiotic residues from various sources, including point sources (domestic and hospital effluents, industrial, and wastewater treatment plant (WWTP) discharges) and diffuse sources (runoff from agriculture and aquaculture areas and informal settlements) [3]. Depending on the environmental persistence and spectrum activity of specific compounds, antibiotic residues from these sources may directly enter river systems. Rivers are critical components of ecosystems, serving various purposes such as water supply for drinking and irrigation purposes. Therefore, the contamination of rivers with biochemical compounds, particularly antibiotics, poses risks to the biosystem and human populations.

Monitoring and assessing this type of pollution is challenging due to the complex mixture and interactions of pollutants. Antibiotics in the environment have been found to exist as conjugates and metabolites of their original forms, which can sometimes be more toxic than the parent compounds [4]. As such, studies focusing on the occurrence of the original forms of antibiotics are crucial for investigating the extent of pollution in the aquatic environment. Although the detected antibiotic concentrations may not immediately raise the alarm, their presence has been associated with the proliferation of antibiotic-resistant microbes [1]. These trace-level antibiotic residues from various sources can pose potential risks to ecosystems and downstream users [5,6,7].

Wastewater treatment plants collect and receive waste discharges from various sources, which could consist of antibiotic residues either deposited from domestic sources as unmetabolized compounds or through the disposal of unused or expired prescriptions from homes, pharmaceutical industries, or health facilities [8]. However, the complete removal of antibiotics in wastewater treatment plants is not always achieved [9,10]. Antimicrobials and their metabolites in wastewater treatment plant effluents lead to elevated pollution levels and concentrations in the aquatic environment, as most antibiotics eventually end up in surface water [11]. Consequently, higher antibiotic residues are often reported in municipal wastewater effluents and have been detected in receiving river water and sediment in many rivers worldwide [12,13,14,15,16,17,18]. In addition, WWTPs are reported as the primary sources for disseminating resistance genes into the aquatic environment [19,20,21,22].

Numerous studies have reported the emergence of antibiotic resistance genes [19,20,21,22,23,24,25]. Antimicrobial resistance in surface water has also been observed in South Africa [19,21,26,27,28]. Abia et al. [29] identified eight Vibrio species genes with resistance to erythromycin (100%), tetracycline (50%), and penicillin g (70%) in wastewater effluent discharged into the Thembisa River, Eastern Cape Province, South Africa. Studies on antimicrobial resistance have reported genomic resistance of Escherichia Coli to tetracycline in the Msunduzi River [30] and in estuaries in Durban, South Africa [26]. The emergence of these new resistance genes could pose risks to human health and aquatic organisms throughout the food chain. Despite these challenges, research on antibiotic pollution in Africa remains scarce. Consequently, the continuous assessment and quantification of therapeutic antibiotics in the aquatic environment are essential for developing strategies to mitigate their impact on the environment and human health.

The detection of antibiotics in the aquatic environment has been a research interest, leading to the development of various detection methods. Given the array of techniques available for separating and analyzing different components in environmental samples and matrices, liquid chromatography–mass spectrometry (LC-MS) has emerged as a method for analyzing antibiotics in water samples [4,31,32,33]. LC-MS is known for its suitability for analyzing organic compounds, offering rapid separation and analysis in water samples [34,35]. LC-MS boasts high sensitivity and selectivity and is well suited for detecting and analyzing trace-level antibiotics in surface waters, down to parts per billion (ppb).

Studies from different parts of the world [36,37,38,39,40] have reported the presence of antibiotics in surface water. The occurrence and fate of antibiotics in the environment have been extensively studied in Asia, Europe, and the USA, while limited research has been conducted in Africa [41,42]. While South Africa has been the primary location for studies on the African continent regarding antipyretic, antiepileptic, antipsychotic, anti-retroviral, and various other drugs [11,13,43,44,45,46,47,48], there has been a notable lack of focus on investigating the presence and distribution of antibiotics employed for therapeutic reasons against bacterial infections in densely populated rivers. Also, the sources of these antibiotic residues in the river and their ecological impact on the aquatic environment have not been thoroughly explored.

Studies on the Umgeni River in South Africa have reported the presence of some antibiotic drug residues in surface water, with concentrations generally lower than 10 µg/L [12,49]. These reports have emphasized the higher contribution of antibiotic residues to the Umgeni River from its tributary, the Msunduzi River. Furthermore, other studies have reported the detection of antibiotics in the Msunduzi River [11,50]. The Msunduzi River receives drainage from diverse regions with distinct land use patterns, resulting in the potential introduction of numerous antibiotics into the river. The production and distribution of antibiotic pollution in the river are influenced by human and land use practices. Therefore, the effective monitoring and control of these antibiotic residues requires an understanding of their temporal and geographical occurrence. However, the sources of these antibiotic residues in the river and their ecological impact on the aquatic environment have not been thoroughly explored [51]. The probability of environmental hazards expected to occur due to a specific antibiotic compound in the aquatic ecosystem can be assessed through screening level risk characterization, comparing the detected concentration in the river with the no-effect concentration (threshold level) of the antibiotic.

This study, therefore, aims to investigate the source, occurrence, persistence, and ecological risk of selected therapeutic antibiotic compounds in the Msunduzi River and its major tributaries in KwaZulu Natal, South Africa. Specifically, four target antibiotics representing four therapeutic classes, Tetracyclines (tetracycline), Macrolides (erythromycin), Sulphonamides (sulfathiazole), and β-lactams (penicillin g), have been selected and investigated. Despite the previous examination of tetracycline (TTC) and erythromycin (ERY), there has been no prior investigation into the presence of sulfathiazole (STZ) and penicillin g (PNC) in South African waste and surface water.

## 2. The Study Area

**Figure 1:**
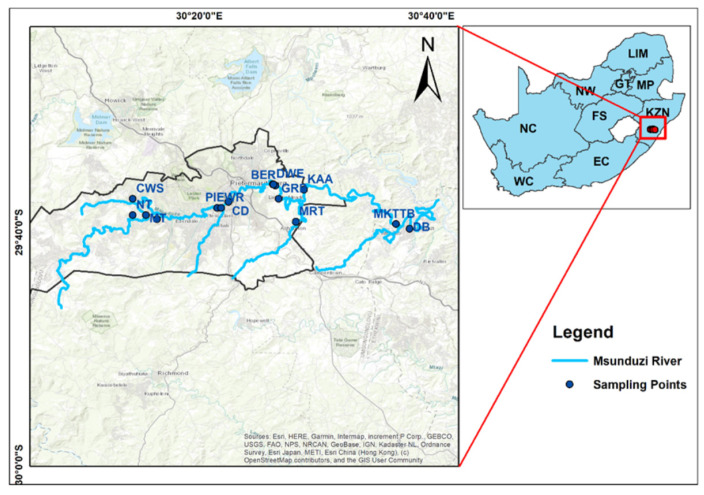
Location map of the sampling points along the Msunduzi River.

Surface water samples were collected from 16 sampling sites (Figure 1) strategically chosen to represent various land use activities, including industrial, agricultural, residential, urban parks, and WWTPs along the Msunduzi River catchment. The Msunduzi River flows through Pietermaritzburg, the capital of the KwaZulu-Natal province in South Africa, serving as the primary drinking water source for the Msunduzi Municipality.

The Msunduzi River catchment is characterized by a densely populated area and is, therefore, affected by various anthropogenic activities and developments [12]. The river drains suburban villages, agricultural areas, municipality WWTP effluent discharge sites, landfills, and informal settlements. The river catchment comprises tributaries and small streams that pass through grazing fields, industrial parks, commercial farming sites, and informal settlements with poor sanitation and inadequate wastewater treatment facilities, which may cause high concentrations of the identified antibiotics [52].

## 3. Materials and Methods

### 3.1. Sample Collection

Three extensive field campaigns were conducted during the spring, autumn, and winter seasons from September 2022 to May 2023. The samples collected encompassed surface water along the Msunduzi River and its tributaries, wastewater effluents from a WWTP, and the immediate injection point of the WWTP discharge into the river. The coordinates and descriptions of the sampling sites are detailed in Table 1.

Preliminary sampling was conducted as a control measure to establish nutrient and pollutant loading at specific sites. Selected parameters were measured in situ using a portable Hanna multi-parameter field probe to maximize data accuracy and ensure data integrity. The physical and chemical characteristics of the sampling sites throughout the sampling period are outlined in Table 2.

Samples were gathered from the 16 selected sites using sterilized 500 mL amber glass bottles. These bottles underwent thorough cleaning, including washing with dDynaChem soap and rinsing with tap water and Milli-Q ultra-pure water. Subsequently, they were washed with acetone to eliminate polar and nonpolar compounds. The sample bottles were sterilized in a steam sterilizer at 125 °C for 15 min, and air steam laminar flow was used to prevent biotic transformation due to microbial and enzymatic activities. Duplicate samples (*n* = 2) were collected from each site at a 1–2 cm depth from the water surface. Each sample bottle was covered with aluminum foil and bottle caps after collection. The samples were then stored in ice boxes at appropriate temperatures, transported to the laboratory for testing, and stored in a dark, cold room at 4 °C before undergoing solid phase extraction (SPE).

### 3.2. Chemicals and Reagents

All antibiotic standards used were procured from Merck (Modderfontein, South Africa). The solubility, chemical formula, octanol-water partition coefficient (Kow), and their molar mass are provided in Table 3. HPLC-grade acetic acid (95%), methanol (99.8%), and acetone (98.5%) were acquired from Sigma-Aldrich (Merck, Modderfontein, South Africa). Whatman filter paper with a 0.45-µm filter diameter was obtained from Sigma-Aldrich (Merck, Modderfontein, South Africa). Laboratory reagent water from a water purification system, specifically Milli-Q ultra-pure water (18Ω), was used. Hydrophobic-Lipophilic Balance (HLB) SPE cartridges (Oasis PRIME HLB 6CC, 20 mmPE, 60 mg, 5 mL) were sourced from Waters Microsep, Pty Ltd. (Cape Town, South Africa).

### 3.3. Stock Solutions

An individual crystal of 10 mg was weighed from each test analyte reagent (TTC, PNC, STZ, and ERY). By dissolving the weighted crystal in 10 mL of a 50v:50v mixture of methanol and milli-Q water, a stock solution of 1000 mg/Lwas prepared. The stock solution was stored in a dark, cold room at a temperature of 4 °C until extraction. Multi-standard solutions for different concentrations were prepared for calibration, utilizing methanol and dilutions from the stock solution.

### 3.4. Sample Extraction

Samples were filtered using 0.45-µm Whatman filter paper (Whatman, Kent, England), after which the Oasis HLB SPE cartridges were used to extract the targeted analytes [34,35,53,54]. The SPE Supeclo manifold (Sigma-Aldrich, Eschenstr, Germany) and HLB SPE cartridges were used to extract the sample. The SPE cartridges were conditioned with 5 mL methanol and 5 mL milli-Q ultra-pure water at 1 mL/min before loading the samples. The pH of the filtered samples was adjusted to a pH of 4 using acetic acid. A total of 400 mL of the pH-adjusted samples was loaded into the conditioned cartridges and allowed to pass through, maintaining the flow rate at 2 mL/min. The solid phase extract was left for 30 min after −70 kPa manifold vacuum drying. The analytes were then eluted with 10 mL of methanol and 5 mL of n-hexane/acetone, each at a flow rate of 2 mL/min. The elutes were dried under a manifold vacuum before being reconstituted in 1 mL of methanol.

### 3.5. Method Validation

The limit of detection (LOD) and the limit of quantitation (LOQ) were calculated using 3 and 10 times the signal-to-noise ratio obtained from the chromatographic analysis. As a quality control measure, a recovery study of the analytes was conducted with the same parameters as those for the samples. Water samples of 500 mL were spiked with the analytes at a spike concentration of 0.01 to 1 mg/L. Three replicates of the recovery study and sample analysis were performed to gauge the method’s reproducibility. The recovery was determined from the concentration differences between the spiked and unspiked samples divided by the spiked concentration. Blank samples were prepared as a control sample, and sample analyses were performed.

### 3.6. Antibiotic Separation and Quantification

The liquid chromatography separation was conducted using SHIMADZU LC-MS-2020 (Shimadzu, Kyoto, Japan) with the column Shimadzu Shim-Pack GIST-HP 3 µm C18, 4.6 × 150 mm. The analyte identification and quantification were carried out in the positive and negative ion modes. The mobile phase consisted of 0.1% formic acid in milli-Q water at 30 °C (Mobile phase A) and 0.1% formic acid in acetonitrile (Mobile phase B). The gradient elution method was used, and the mobile phase compositions are presented in Table 4. The column was maintained at 35 °C, and an injection volume of 20µL was employed. The column was allowed to calibrate for 5 min before the next injection. The analysis was conducted for 40 min, and the retention times (R_T_) were between 10 and 24 min for the four detected analytes.

### 3.7. Environmental Risk Assessment

The risk quotients (RQs) method is usually applied to assess the ecological risk of antibiotics following the guidance of the European Commission technical document [55]. Screening level risk assessment for the antibiotics analyzed in this study was performed. The risk quotient is calculated using Equation (1).

(1) $$\mathrm{RQ}=\frac{\mathrm{MEC}}{\mathrm{PNEC}}$$

where the predicted or measured environmental concentration (MEC) of the detected antibiotic is divided by the PNEC values reported for the aquatic species [16,18,56,57]. The predicted no-effect concentration (PNEC) is the concentration at which the antibiotics cause no undesirable effect on the environment and non-target aquatic organisms. The PNEC can be estimated from toxicology test data of aquatic organisms (e.g., algae, fish, protozoa, and crustaceans) based on the minimum inhibition concentration (MIC). The MIC includes the no observed adverse effect level (NOAEL), the lowest observed adverse effect level (LOAEL), and EC_50_ in laboratory studies. EC_50_ is the concentration of measured antibiotics in the aquatic environment that causes an effect on 50% of the exposed aquatic organisms. PNEC is usually estimated by applying an assessment factor (AF) to account for variability and uncertainties in the ecotoxicology data, as shown in Equation (2).

(2) $${\mathrm{PNEC}}_{aquatic\;organism}=\frac{MIC}{AF}$$

In comparison, when MEC is greater than PNEC (i.e., MEC/PNEC > 1), risk is suspected, and the observed antibiotics are considered to have an ecological risk depending on the sensitivity of the ecological receptor. RQ < 0.1 is assumed that risk is insignificant, 0.1 ≤ RQ ≥ 1 is low risk, 1 ≤ RQ ≥ 10 is moderate risk, and RQ >10 is high risk. The PNEC data used in this study is obtained from [58].

## 4. Results and Discussion

### 4.1. LCMS Detection of Analyzed Antibiotics

**Figure 2:**
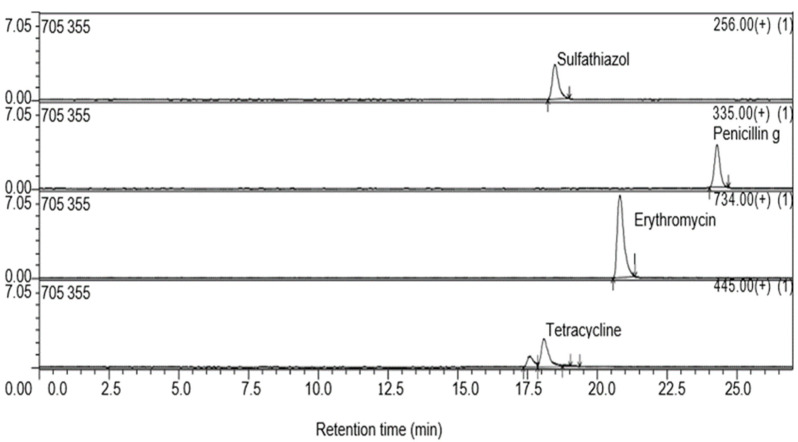
LC-MS analysis of targeted antibiotics.

All the analytes were detected at wavelengths of 254 nm and in the positive ion mode (Figure 2). The mean percentage recovery with standard deviation (SD), LOD, and LOQ are presented in Table 5.

### 4.2. Occurrence and Concentration of Detected Antibiotics

All target analytes were detected in both the surface and wastewater effluent samples. TTC, followed by STZ and PNC, were detected more frequently in most of the sampling sites, while the detection of ERY was less frequent. Further, the detection of the analytes at higher concentrations was observed in samples collected during the spring season than those collected in the autumn and winter seasons. The concentration of the investigated antibiotic analytes from both surface and wastewater effluent samples is presented in Table 6. The concentration of the targeted antibiotics from the wastewater effluent samples was in the range of 138.03–1756.51 ng/L for TTC, <LOQ for PNC, 120.74–5613.58 ng/L for STZ, and 52.14–142.63 ng/L for ERY. High antibiotic concentrations were observed from the WWTP effluent point and discharge into the Msunduzi River. The maximum concentration of STZ (5613.58 ng/L), followed by TTC (1756.51 ng/L) and ERY (142.63 ng/L), was observed during the spring season from WWTP effluents while PNC was detected as below the limit of quantitation, i.e., 143.98 ng/L. In the river water samples, the levels of the analyzed antibiotics were found in the range of 158.42–1290.43 ng/L for TTC, 143.98–503.30 ng/L for PNC, 112.68–1151.25 ng/L for STZ, and 52.14–106.63 ng/L for ERY. The maximum concentration of analytes was observed 1 km downstream of the WWTP effluent discharge with TTC (1290.43 ng/L) and STZ (1151.25 ng/L). However, the maximum concentration of PNC (503.30 ng/L) and ERY (106.63 ng/L) within the Msunduzi River water sample was observed in samples collected at its tributaries, namely, the Wilgerfontein River (upstream tributary located in the city of Pietermaritzburg) and the Mshwati River (downstream tributary), respectively.

The detection of these analyzed antibiotics at a relatively higher concentration in the study area is invariably linked to the inherent characteristics of each antibiotic and the prevailing human activities and land use practices in the studied catchment. For instance, STZ is an antibiotic known for its broader use in animal husbandry as a feed additive and for treating severe infections in human medicine. However, STZ cannot be completely metabolized in the liver and kidneys, and is excreted either in its unchanged form or as metabolites [42]. This is confirmed as results from our analysis show that STZ is found more frequently in the Msunduzi town sample locations MT, CWS, and BMT, which have a significant informal settlement population and are suburban regions. Further, it was detected in commercial livestock farming areas (KAA and MRT), which have extensive grazing for breeding and meat production, where its detection in this area could be attributed to its use by veterinarians.

Furthermore, STZ is known to have poor sorption properties, resulting in a low removal rate in WWTP (low sorption to treatment sludges) [59]. It persists at high concentrations in the pore water of sediments for more than a half-life of 50 days. Under normal environmental conditions, STZ has a relatively lesser attenuation rate than other antibiotics, such as tetracyclines and macrolides [42,60]. Thus, its presence in the environment poses risks to the aquatic biosystem as its degradation is a slow process.

Similarly, penicillins are among the most influential families of antibiotics used in veterinary and human medicine. In this study, PNC was detected in the range from 143.98 to 503.30 ng/L in the river water, whilst the detection in the treated wastewater effluent samples was observed below the quantitation limit. PNC is a weak base and hydrophilic [61], and has a poor stability of the b-lactam ring in the aquatic environment. The b-lactam ring hydrolyses easily under acidic and alkaline conditions or by reaction with weak nucleophiles such as water and metal ions or by widespread enzymes in bacteria in the same way as acidic hydrolysis [61].

Therefore, its detection in surface water samples could be a real-time occurrence. Moreover, the low-level detection of PNC in the wastewater effluent sample is because PNC has a high biodegradation rate in various solid matrices and hydrolysis [61,62]. Higher attenuation of penicillin is related to hydrolysis than the other attenuation mechanisms. The unstable structure of β-lactam ring is highly affected by pH and heat, and it can be converted to Penicilloic acid, Penicilloaldehyde, Penicillamine, Penicilloic, and Isopenillic acid [4]. As a result, the presence of PNC in the samples is low due to its swift transformation in the environmental media (through the easy hydrolysis of the b-lactam ring).

TTC is an antibiotic administered for both human and veterinary usage. Its use is sometimes associated with pain relief among people involved in high-energy physical activity and within all population groups, including the lower income earner groups, which is the case with informal settlement dwellers. Further, tetracyclines are considered the most prevalent antibiotics because they are primarily used as feed additives for animal farming [42].

Thus, TTC was detected in concentrations ranging from 137.07 to 684.11 ng/L in the tributaries (BMT, PIE, WR, and MRT), industrial effluent (PIE), and suburban informal settlement areas (MT and DB). The results indicate a high possibility of TTC being applied in livestock production being transported into the water system through wastewater effluent discharge and other diffused sources, such as runoff from informal settlements. Further, a high concentration of TTC, 1756.51 ng/L, was detected in the wastewater effluent taken during the spring season. This high concentration can be attributed to TTC’s persistence in conventional wastewater and sludge treatment systems [63,64,65]. However, it is observed that the concentration dropped to 1290.43 ng/L, 1 km downstream of the effluent discharge point. This is likely due to the dilution, sorption, and hydrolysis, which causes antibiotic removal from the water column [63,65,66].

Tetracyclines are hydrophobic compounds and sorb into the sediment while being degraded to a smaller extent by photolysis [4]. The low concentration of TTC in the sampling points in the surface water of the river can be attributed to its high affinity to adsorption components through its multiple functional groups and less desorption affinity to different sediments [67].

ERY concentration in this study was observed as ranging from 52.14 to 106.63 ng/L in surface water and 142.63 ng/L in the treated wastewater effluent samples. It was detected in the main Msunduzi River, in the tributaries (BMT and MRT), and from industrial effluent (PIE). Therefore, it can be noted that human activities (e.g., human medication in hospitals and medicine production industries) are major sources of ERY along the Msunduzi River. ERY is stable for hydrolysis and sorption, while they are sensitive to photodegradation by the mechanism of cladinose ring cleavage. ERY is also degraded by the action of bacterial species (*Ochrobactrum* sp. Strain) through the transformation of depyranosyloxy [4,68]. This could cause the low-level detection of ERY in the wastewater effluent samples. Further, ERY was observed more frequently during winter than in spring (Table 6). Thus, it is noted that photolysis has a role in its occurrence in the environment. However, the observed concentration of the analytes may not only be attributed to anthropogenic activities but can also be contributed from naturally producing bacteria. For instance, the Actinomycetes group and Streptomycetes have often been reported for the contribution of b-lactam and TTC load to the environment [69,70,71]. A study [72] showed that polyketide synthase genes (PKS I/PKSII) are responsible for the natural production of macrolide antibiotics, such as ERY and tylosin. 

Further, the presence of anions and metallic ions may play a role in the occurrence and persistence of antibiotic residues. The abundance of Cl^−^, NO_2_^−^_,_ and NO_3_^−^ may inhibit the photodegradation of STZ [72,73]. Tang et al. [74] reported that NO_3_^−^ inhibits the indirect photodegradation of STZ by reducing the steady-state concentration of excited reactive intermediates, which are sensitizers for removing the compound. NO_3_^−^ is an important Hydroxyl radical Oxidant (HO) photosensitizer source. The photolysis of nitrate generates nitrite ion, which plays a decisive role by masking HO sensitization [74,75].

Br^−^ has been documented to enhance the degradation of TTC, while Cl^−^ has been reported to potentially hinder TTC degradation by scavenging the reactive form of SO_4_^2−^ forming inactive chlorine species [76]. Tetracyclines are frequently found at low concentrations in the aquatic environment, primarily due to cations like calcium facilitating their precipitation and accumulation in solids/sediments [76,77].

### 4.3. Comparison of the Result with Previous Studies

The concentration of TTC detected from the WWTP effluent samples in this study is comparable to previous studies in South Africa. Kanama et al. [13] reported the detection of TTC from two WWTP effluents in the North Waste province of South Africa, reporting TTC concentrations of between 520–1430 ng/L in WWTP A and 480–3220 ng/L in WWTP B. In addition, the concentration of TTC was slightly lower than those found by Agunbiade and Moodley [78], who reported a 3700 ng/L concentration of TTC from Northern WWTP effluent discharging into the Umgeni River, Kwazulu Natal province, South Africa.

The detected concentration of TTC in the surface water samples aligns with previous investigations of the Umgeni River, ranging from 100 to 3100 ng/L, as reported by Agunbiade and Moodley [78]. However, the TTC concentration was lower than that documented by Fatoki et al. [79], where the maximum TTC concentration in the country is between 3780 and 4880 ng/L in surface water samples from Cape Town, Eastern Cape province, South Africa.

The concentration of ERY from this study is comparable to a prior investigation by Matongo et al. [80], who reported a concentration of 160 ± 730 ng/L of ERY from an effluent sample of Darville WWTP. However, it was lower than the concentration reported by Agunbiade and Moodley [78], who measured a concentration of 1300 ng/L of ERY from a WWTP effluent in Kwazulu Natal, South Africa.

Regarding surface water concentrations, Matongo et al. [80] reported a concentration of 60 ± 13,560 to 240 ± 9930 ng/L of ERY from surface water samples in the Msunduzi River. This contrasts with the ERY concentration detected in this study, which is much lower. However, the ERY level in surface water reported in this study is comparable to that of Agunbiade and Moodley [78] with concentrations of 580 ng/L in the Umgeni River.

Furthermore, the level of ERY obtained in this study is comparable to the levels detected by Vumazonke et al. [81] for Buffalo River (84–263 ng/L), Bloukrans River (164–744 ng/L), and Tyhume River (11–118 ng/L). However, it is slightly lower than the concentrations found in Swartkops River (35–11,800 ng/L), analyzed during spring seasons from different rivers in Eastern Cape province, South Africa [81].

The levels of TTC and STZ detected in this study are comparable with studies reported for wastewater effluents from other countries, as shown in Table 7. However, ERY concentrations reported from other countries are higher than those detected in this study, where 275 ng/L was reported in Egypt [82] and 1187 ng/L in Tunisia [83]. Additionally, the levels of PNC and STZ reported in this study are lower than those reported from Canada and the USA. Concentrations of ERY, PNC, and STZ reported from Korea are higher than those discovered in this study.

Moreover, in the surface water, the ERY, TTC, and PNC levels reported in this study are slightly higher than those reported in Australia, China, and the USA. At the same time, they are comparable with those reported in Nigeria, Egypt, Pakistan, and Korea. The level of antibiotics detected in this study was comparable to other developing countries from Africa and Asia, both in the waste and surface water. However, the level of antibiotics in this study was slightly higher than those reported from surface water samples from developed countries. These observed differences might be related to various factors, primarily antibiotic consumption, living standards, waste disposal regulation, and wastewater treatment technologies.

From these studies, it can be deduced that the concentration of antibiotics in the surface water of South Africa is higher than that of global averages. Nevertheless, considering the varying efficiency in removing antibiotics in the WWTP facilities, the above studies may not be adequate for drawing definitive conclusions about their comparative persistence in surface water. Therefore, more investigations are needed. Further, the environmental monitoring of waste disposal regulations and the application of advanced technologies are essential for sustainable aquatic environment management.

### 4.4. Spatial Distribution and Seasonal Variation of Detected Antibiotics

The targeted antibiotics were detected more frequently during the spring (36.4%) than the autumn (30.3%) and winter (33.3%) seasons in the collected samples. This might be linked to antibiotic detection being enhanced during the dry water period. The increased detection of antibiotics throughout the spring (dry period) can be ascribed to antibiotic persistence due to a lack of dilution by rainfall. Furthermore, antibiotic persistent sites (e.g., dissolved organic materials, algae, and suspended solids) are more abundant during dry than rainy seasons. TTC and STZ were also detected more frequently than PNC and ERY during both seasons.

**Figure 3:**
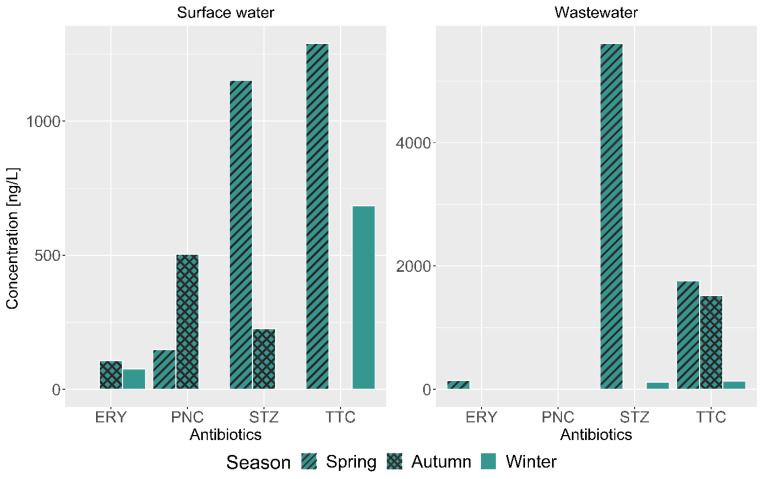
Comparison of antibiotic detection during the spring, autumn, and winter seasons for wastewater effluent and surface water samples along the Msunduzi River.

Further, the level of TTC and STZ was slightly higher during spring (dry water period) than in autumn and winter, as seen in Figure 3. Compared to the other examined antibiotics, ERY was detected at a low level below 150 ng/L and, in some instances, undetected. This can be attributed to the pattern of antibiotic usage, where there is less consumption of ERY during the dry period than in the wet season (autumn).

The levels of antibiotics found during autumn, characterized by the wet season, are lower than those observed in spring and slightly higher than in winter (excluding TTC in the surface water). However, a noteworthy event occurred a week before the sample collection on 19 March 2023, where a significant rainfall of 109.22 mm was recorded. This rainfall event could potentially lead to decreased antibiotic concentrations in the river. Flooding may result in lower antibiotic concentrations due to the dilution and transport of residues becoming more prevalent in the river.

This phenomenon may be attributed to the dilution of antibiotic residues accumulated in the water channel, stagnant water, and sediment, which are washed off by runoff. Consequently, the flooding incident could contribute to decreased antibiotic levels in the river during the autumn season.

**Figure 4:**
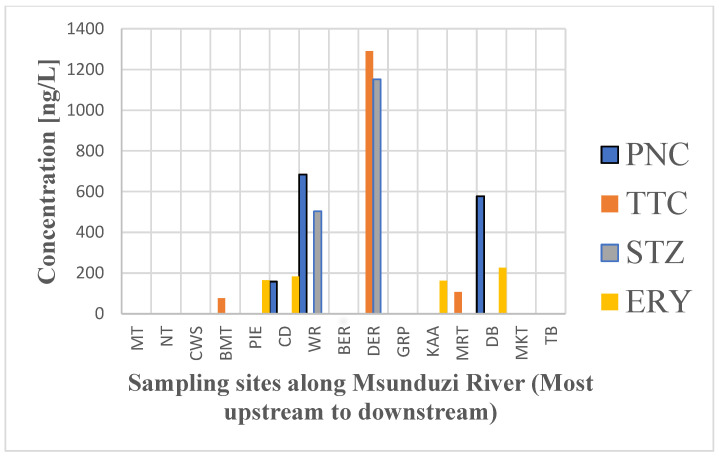
Spatial distribution of detected antibiotics along the Msunduzi River from the most upstream (MT) to downstream (TB).

The spatial variability of antibiotics from the results shows that anthropogenic activities like industries, aquaculture, commercial farms, grazing land, and informal settlements have a significant impact on the upper reach of the Msunduzi River (MT, NT, CWS, BMT, PIE, CD, WR, BER, DWWE, and DER). These human activities produce a sizable amount of antibiotic residue at the river’s upper reach, exhibiting a significantly higher detection and concentration of the targeted antibiotics than the lower reach (Figure 4).

PNC residues were detected at the Mabane tributary streams (BMT) and Wilgerfontein tributary (WR). STZ was also observed in the Mabane tributary (BMT), Wilgerfontein tributary (WR), and Msunduzi River before the Darville WWTP effluent release. The Kwapata and Mvubukazi tributary streams and the Wilgerfontein tributaries drain waste from industrial effluents and household waste, contributing to the Msuduzi River’s antibiotic residue. Further, MRT, KAA, and MKT drain from lands majorly occupied by agricultural farms and suburban developments. Thus, these target antibiotic residues from the tributaries might be related to antibiotic usage, which could cause continuous antibiotic residual sources in the Msunduzi River.

The antibiotic pollution of the Msunduzi River is primarily caused by the effluent from the Darville WWTP, as shown in Figure 4. Effluent from the Darville wastewater treatment plant is found to be a hotspot and primary source for high antibiotic residual contamination in the river. Therefore, the proper treatment and monitoring of waste discharge releases from the industries and municipal wastewater treatment plants are essential. In addition, it must be noted that similar to the temporal variations, the location (the spatial distribution) is an essential factor in characterizing antibiotic pollution in a river.

### 4.5. Risk Assessment

Ecological risk assessment was conducted for the antibiotics detected and quantified in this study (Table 8). The antibiotic toxicity threshold, sourced from the relevant literature [18,58,93,94], was determined based on the no observed effect concentration (NOEC), the lowest observed effect concentration (LOEC), and the concentration at which 10% of the organisms are affected (EC10) [18]. The average concentration of analyzed antibiotics in the surface water was considered as the minimum effect concentration (MEC). The minimum inhibition concentration (MIC) was employed for algae, fish, and daphnids, utilizing assessment factors as outlined in [58].

Based on the assessment, only TTC poses a high risk for fish, and no adverse ecological risks were evident from the other antibiotics investigated in this study. ERY presents a moderate risk for algae (RQ = 2.286). Additionally, TTC exhibits a low risk for algae and daphnids. STZ and PNC show no observed ecological impact on any organism. It is important to note that the occurrence of these antibiotics in surface samples at trace levels may not pose immediate risks; however, their continuous presence could harm the ecology. Moreover, the toxicity sensitivity of a specific species may vary based on its genomic characteristics from one country to another. Therefore, future studies should include specific investigations into ecological risk assessment.

## 5. Conclusions

This study comprehensively investigates the presence of four antibacterial drugs and their potential ecological implications in the Msunduzi River. The investigation reveals the widespread occurrence of selected antibiotics along the Msunduzi River in the KwaZulu-Natal province of South Africa, with TTC and STZ emerging as predominant contaminants, demonstrating both frequent detection and higher concentrations in both wastewater effluent and river surface water when compared to other antibiotics investigated in this study.

Compared with the dry season, lower concentrations of antibiotics in the river water and treated wastewater effluent were found in the wet season, probably due to the dilution effect of high water flow. The level of antibiotics detected in the surface water was higher than in developed countries. In contrast, it was comparable in developing countries from Africa and Asia, both in the waste and surface water.

Notably, the study’s findings underscore the critical role of wastewater treatment plant effluent in elevating antibiotic residue levels within the Msunduzi River. Moreover, the presence of these antibiotics in the tributaries and their seasonal variability strongly implicates anthropogenic activities in driving antibiotic contamination into the river. While the input of antibiotic residues from WWTP effluent remains prominent, our investigation identifies additional contributors, including commercial farming, industrial discharges, and informal settlements in the catchment and proximity to the river banks, disseminating antibiotic residues in the river.

Ecological impact risk assessment was performed to deliver insight into the prioritization of antibiotics to assist future regulation plans. TTC was identified as posing a significant risk to fish. In contrast, the infrequent presence of ERY, found at low levels, was associated with a moderate risk to algae. It was also observed that the risk to the ecological species may not necessarily attributed to the frequent and high-level detection of antibiotics but also to the persistence of the antibiotics and sensitivity of the exposed species. Therefore, the proper treatment and monitoring of waste discharge releases from the industries and municipal wastewater treatment plants, enhancing the efficacy of treatment facilities in removing organic pollutants, continually monitoring their optimal operation, and raising awareness regarding responsible waste discharge practices are vital for the preservation of river ecosystems.

In conclusion, our findings underscore the importance of further research to gain a nuanced understanding of antibacterial drug usage, disposal management, and the comprehensive evaluation of their ecological impact to develop and implement best practices for mitigating potential risks.

## Figures and Tables

**Table 1:** Coordinates of sampling sites along the Msunduzi River, South Africa.

S/No	South	East	Location	Abbreviation	Description
1	29.64169	30.25631	Msunduzi Town	MT	
2	29.64169	30.23749	Nqabeni tributary	NT	
3	29.61837	30.23751	Car wash stream	CWS	
4	29.64755	30.27233	Below Mabane tributary	BMT	
5	29.63135	30.35887	PMB Industrial effluent	PIE	Kwapata and Mvubukazi streams
6	29.6226	30.375	Camps Drift	CD	
7	29.63136	30.36443	Wilgerfontein River	WR	
8	29.59909	30.44254	River water before effluent release	BER	
9	29.59725	30.43886	Darville WWT effluent	DWWE	
10	29.3549	30.2625	River water after effluent release	DER	1 km downstream of the effluent discharge
11	29.61822	30.44724	Gripthorpe	GRP	Bayne’s Spruit tributary
12	29.60502	30.48338	Kayeni Agricultural area	KAA	Ashburton Commercial Farm
13	29.65112	30.47177	Mpushini River tributary	MRT	
14	29.6613	30.63542	Duzi Bridge	DB	Farm and Informal settlement
15	29.3932	30.3709	Mshwati River tributary	MKT	Informal settlement
16	29.3932	30.3657	Table mountain	TB	Near the joining of Umgeni River

**Table 2:** Physicochemical characteristics of the river during the sampling.

Parameter	MT	NT	CWS	BMT	PIE	CD	WR	BER	DWWE	DER	GRP	KAA	MRT	DB	MKT	TB
Spring																
pH	7.0	7.7	5.8	6.1	7.7	8.1	7.7	7.7	6.7	7.5	7.6	7.8	7.6	7.9	7.2	7.7
T (°C)	20.4	24.1	18.8	14.3	16.4	17.9	15.4	15.9	22.4	23.3	22.0	22.7	24.5	20.0	28.9	22.4
EC (µs/cm)	8464.0	2.0	14.0	77.0	88.0	90.0	93.0	151.0	232.0	415.0	236.0	426.0	415.0	243.0	265.0	586.0
TURB (FNB)	4232.0	1.0	7.0	38.0	44.0	45.0	47.0	75.0	116.0	207.0	118.0	213.0	207.0	121.0	133.0	293.0
ORP (mv)	168.6	293.5	264.6	233.5	195.9	191.9	205.4	32.5	120.2	142.6	109.1	139.3	142.6	147.8	132.2	137.5
F (ppm)				0.1			0.1								1.1	
Cl^−^ (ppm)	11.3	44.5	10.7	60.7	23.4	25.6	35.5	42.6	88.0	29.4	41.3	8.7	195.5	10.7	419.1	41.7
NO_3_^−^ (ppm)	2.0	5.4	3.6	7.5			1.0	1.4	3.3	3.4	3.1	1.9	1.6	1.8	17.2	7.8
SO_4_^2−^ (ppm)	3.1	21.1	5.5	19.9	20.7	17.0	19.4	24.1	41.2	15.6	21.8	2.7	28.7	3.0	111.0	17.9
PO_4_^3−^ (ppm)									0.1							
Autumn																
pH	7.4	8.3	8.1	8.2	7.6	7.5	7.6	7.6	7.6	8.0	7.8	7.6	7.7	7.6	7.7	7.9
T (°C)	18.1	19.6	23.1	22.7	22.9	24.3	24.7	25.9	27.7	25.1	23.2	23.4	23.8	23.5	23.5	23.8
EC (µs/cm)	70	90.0	96.0	89	96	105	413.0	247.0	547.0	413	253.0	252.0	669.0	253	871.0	230.0
TURB (FNB)	3.1	4.5	5.8	28.6	49.3	52.5	109.7	323.4	34.1	0.0	77.2	71.0	161.9	94.8	134.2	11.5
ORP (mv)	309.0	201.6	222.7	223.1	252.2	268.4	66.7	185.4	196.5	171.1	180.3	202.2	187.8	178.8	188.1	195.6
F (ppm)															0.6	
Cl^−^ (ppm)	5.4	8.6	9.8	10.1	19.7	21.4	36.0	21.0	63.8	25.1	23.6	24.6	100.5	29.4	329.5	21.9
NO_3_^−^ (ppm)	3.0	5.5	5.7	6.2	8.6	6.8	6.5	6.8	4.7	6.5	6.4	7.8	0.3	9.6	3.8	2.1
SO_4_^2−^ (ppm)			2.3		2.5	2.0	3.5	2.5	6.8	3.0	2.7	2.7	3.7	3.1	30.7	3.1
PO_4_^3−^ (ppm)																
Winter																
pH	6.0	7.2	7.0	7.2	6.9	7.0	6.9	7.2	7.6	7.4	6.4	7.0	6.7	6.6	6.9	7.1
T (°C)	12.2	16.1	15.5	14.3	14.5	16.1	15.5	15.5	16.7	19.6	23.3	18.0	28.9	26.5	28.0	15.9
EC (µs/cm)	119	110	111	88	90	98	39	229	240	388	234	245.8	295	273	274	125
TURB (FNB)	46.2	63.5	29.3	210.8	6.0	133.5	82.0	30.4	12.0	17.0	11.6	204.4	60.5	15.4	9.4	0.0
ORP (mv)	301.0	344.7	327.9	293.7	346.6	324.0	305.5	280.4	246.6	262.7	226.3	2.2	241.3	273.2	254.9	244.2
F (ppm)													0.1		1.2	
Cl^−^ (ppm)	12.4	11.1	13.0	14.5	35.1	31.3	48.5	30.7	88.1	41.0	38.0	40.3	176.9	69.3	493.5	45.8
NO_3_^−^ (ppm)	7.3	6.0	7.3	6.9	7.9	10.9	16.4	12.8	16.2	15.3	12.1	11.9	2.7	13.5	22.7	12.3
SO_4_^2−^ (ppm)	2.3	2.4	6.0	3.3	15.4	11.9	24.1	12.5	40.9	17.5	18.0	17.1	31.4	25.2	37.4	20.4
PO_4_^3−^ (ppm)									3.1							

EC is the electrical conductivity, TURB is the water turbidity and ORP is the oxidation reduction potential which measures the ability of the river to cleanse itself.

**Table 3:** Targeted antibiotics and their physical properties.

Antibiotics	Log Kow (pKa)	Solubility (mg/L)	Chemical Formula	Molar Mass (g/mol)
ERY	3.06 (8.89)	4.2	C_37_H_67_NO_13_	734
PNC	1.83 (2.74)	210	C_16_H_18_N_2_O_4_S	334
STZ	0.05 (7.2)	2370	C_9_H_9_N_3_O_2_S_2_	255
TTC	−1.37 (3.30)	231	C_22_H_24_N_2_O_8_	444

Octanol-water partition coefficient (Kow) measures the relative solubility of a compound in octanol compared to water.

**Table 4:** LC-MS parameters and gradient composition used during the quantification of antibiotics.

Chromatograph	SHIMADZU LCMS-2020		
Column	Shim-Pack GIST-HP 3 µm C18		
Injection Volume (µL)	20		
Temperature (°C)	35		
Flow rate (mL/min)	0.25		
Gradient Composition	Time (min)	%A	%B
	0	95	5
	25	10	90
	27	10	90
	32	95	5
	37	95	5

**Table 5:** Mass detection and separation parameters for the analysis of target analytes.

Compound	Linear Range (ng/L)	Recovery ± SD (%)	R_T_ (min)	Precursor Ion (*m*/*z*)	LOD (ng/L)	LOQ (ng/L)	R^2^
TTC	100–10,000	93.2 ± 4.4	18	445 [M + H]^+^	40.22	137.07	0.996
PNC	10–10,000	81.3 ± 1.7	24	335 [M + H]^+^	43.19	143.98	0.892
ERY	1–100,000	90 ± 2.1	21	735 [M + H]^+^	15.64	52.14	0.997
STZ	10–10,000	91.3 ± 3.6	18	256 [M + H]^+^	37.4	112.68	0.961

**Table 6:** Concentration of analyzed antibiotics (ng/L) at each sampling site along the Msunduzi River.

Antibiotics	MT	NT	CWS	BMT	PIE	CD	WR	BER	DWWE	DER	GRP	KAA	MRT	DB	MKT	TB
Spring																
PNC	ND	ND	ND	ND	ND	ND	<LOQ	<LOQ	ND	ND	ND	ND	ND	ND	ND	ND
TTC	ND	ND	ND	ND	<LOQ	158.42	ND	<LOQ	1756.51	1290.43	ND	ND	<LOQ	577.63	ND	ND
ERY	ND	ND	ND	ND	ND	ND	ND	ND	142.63	<LOQ	ND	ND	ND	ND	ND	ND
STZ	ND	ND	ND	ND	164.64	183.47	ND	ND	5613.58	1151.25	ND	162.88	ND	ND	ND	ND
Autumn																
PNC	ND	ND	ND	<LOQ	ND	ND	503.30	ND	ND	ND	ND	ND	ND	ND	ND	ND
TTC	ND	ND	ND	<LOQ	ND	ND	<LOQ	ND	1519.65	720	ND	ND	ND	ND	ND	ND
ERY	ND	ND	ND	<LOQ	<LOQ	ND	ND	ND	<LOQ	ND	ND	ND	106.63	ND	ND	ND
STZ	ND	ND	ND	<LOQ	ND	ND	ND	ND	<LOQ	ND	ND	ND	ND	226.18	ND	ND
Winter																
PNC	ND	ND	ND	ND	ND	ND	ND	ND	<LOQ	ND	<LOQ	ND	ND	ND	ND	ND
TTC	ND	ND	ND	ND	ND	<LOQ	684.11	ND	138.03	ND	ND	ND	ND	<LOQ	ND	ND
ERY	ND	ND	ND	76.3	ND	ND	ND	ND	<LOQ	ND	<LOQ	ND	ND	ND	ND	ND
STZ	<LOQ	ND	<LOQ	ND	ND	ND	ND	<LOQ	120.74	<LOQ	ND	ND	<LOQ	ND	ND	ND

ND = Not Detected; LOQ = Limit of Quantification.

**Table 7:** Analysis of the levels of antibiotics from this study and earlier investigations reported from other countries.

	Concentration (ng/L)			
Antibiotics	This Study	Other Studies	Country	Citation
Wastewater effluent				
TTC	1756.51	853	Vietnam	[84]
		1420	Korea	[37]
ERY	142.63	275	Egypt	[82]
		1187	Tunisia	[83]
		2350	Korea	[85]
		48,520	Vietnam	[84]
PNC	<LOQ	11	Canada	[86]
		13,500	Korea	[85]
STZ	5613.58	350	USA	[36]
		16	Canada	[86]
		5000	Korea	[87]
		600	Australia	[39]
Surface water				
TTC	1290.43	50	Nigeria	[88]
		138	Tunisia	[83]
		120	Pakistan	[89]
		138	Vietnam	[84]
		430	China	[90]
ERY	106.63	1000	Nigeria	[88]
		61	Egypt	[82]
		741	Vietnam	[84]
		17	China	[90]
		310	Pakistan	[89]
PNC	503.30	668	China	[40]
		250	Australia	[39]
STZ	1151.25	253	Korea	[91]
		4610	Korea	[92]

**Table 8:** Risk quotients from the detected concentration of targeted antibiotics in the Msunduzi River.

Antibiotics	MEC (ng/L)	Organism	MIC (ng/L)	AF	PNEC (ng/L)	RQ
TTC	686.118	Algae	50,000 (NOEC) ^a^	10	5000	0.114
		Fish	500 ^b^		50	11.43
		Daphnids	10000 ^b^		1000	0.571
PNG	503.3	Algae	6.51 × 10^10 b^	1000	6.51 × 10^6^	0.000008
		Fish	2.05 × 10^13 b^		2.05 × 10^10^	0
		Daphnids	1.32 × 10^11 b^		1.32 × 10^8^	0.000004
ERY	91.465	Algae	2000 ^b,c^	50	40	2.286
		Fish	10^8 b^		2 × 10^6^	0.00004
		Daphnids	220,000 ^b^		4400	0.02
STZ	377.684	Algae	1.31 × 10^7^ (NOEC) ^a^	1000	13,100	0.02
		Fish	510^8 b^		500,000	0.0007
		Daphnids	2.2 × 10^8 b^		220,000	0.0017

Each organism was assumed to be exposed to the quantified concentration MEC. MIC is predetermined using a bioassay experiment defining the minimum organism inhibition concentration. Considering the safety factor and the PNEC, the potential risk posed by the antibiotics to the organisms was calculated as a risk quotient. ^a^ [18], ^b^ [58], ^c^ [93].

## Data Availability

Data are contained within the article.
